# Editorial: Educational robotics and competitions

**DOI:** 10.3389/frobt.2024.1394849

**Published:** 2024-04-09

**Authors:** Felipe N. Martins, José Lima, Andre Schneider de Oliveira, Paulo Costa, Amy Eguchi

**Affiliations:** ^1^ Sensors and Smart Systems Group, Institute of Engineering, Hanze University of Applied Sciences, Groningen, Netherlands; ^2^ Research Centre in Digitalization and Intelligent Robotics (CeDRI), Instituto Politécnico de Bragança, Bragança, Portugal; ^3^ Academic Department of Electronics, Federal Technological University of Paraná (UTFPR), Curitiba, Brazil; ^4^ Faculty of Engineering, University of Porto, Porto, Portugal; ^5^ Department of Education Studies, University of California San Diego, La Jolla, CA, United States

**Keywords:** simulation, educational robotics, stem, engineering education, robotics competitions

STEM education endeavors to instill fundamental principles of science, technology, engineering, and mathematics, fostering a passion that propels students toward careers in these fields. Robotics, as a formidable educational tool, goes beyond theoretical learning by immersing students in practical projects that demand problem-solving skills. Its inherently multidisciplinary nature encourages the integration of knowledge from traditionally distinct disciplines, enriching the educational experience.

In the ever-evolving landscape of STEM education, the integration of robotics has proven to be a dynamic catalyst for inspiring students and advancing research in science, technology, engineering, and mathematics. The latest strides in this interdisciplinary field are encapsulated in four articles published in the Research Topic on Educational Robotics and Competitions. These articles shed light on diverse aspects of robotics challenges, from virtual and real-world competitions to classroom applications.

Martins et al. presents a compelling case study of how the challenges posed by the COVID-19 pandemic led to innovative solutions in the realm of educational robotics in general, and the RoboCupJunior specifically. The RoboCupJunior competition is aimed at students between 14 and 19 years old, providing challenges of appropriate difficulty. However, the restrictions on physical gatherings during the pandemic made it impossible for this physical competition to happen. Members of the RoboCupJunior Soccer Organizing Committee, together with volunteers, rose to the challenge by developing SoccerSim, a simulated environment for RoboCupJunior Soccer based on the Webots open-source robotics simulator. This virtual platform not only allowed the competition to take place despite the pandemic but also significantly lowered the barrier to entry, as evidenced by the participation of many teams that had not participated before. The success of SoccerSim at RoboCupJunior Worldwide 2021 suggests that simulated environments can provide an affordable and accessible alternative to physical robotics platforms.

Another article focuses on a competition for more advanced students in the fields of engineering and computer science. Braun et al., and introduces a new competition in the Portuguese Robotics Open, created in line with the Industry 4.0 concept. The competition named RobotAtFactory 4.0 simulates a fully automated industrial logistics warehouse, presenting unique challenges for Autonomous Mobile Robots (AMRs). The paper describes an innovative approach to the indoor localization system for the competition based on the Extended Kalman Filter (EKF) and ArUco markers. The authors tested and compared different innovation methods for the obtained observations in the EKF, validating their approach in a real scenario using a factory floor with the official specifications provided by the competition organization.

Still in the realm of competitions, a new and innovative robotic challenge is described in Domingos et al. In the proposed challenge, legged robots must climb a volcano’s escarpment and collect data from areas with high temperatures and toxic gases, in a simulated volcanic eruption scenario. The paper describes the design and implementation of the simulated scenario of the volcano ramp, the rules proposed for the competition, and the conception of a robot prototype. It later discusses the performance of teams invited to participate in the challenge in the context of Azorean Robotics Open, the Azoresbot 2022. Then, it reports the feedback from the participants, who found the challenge exciting, challenging, and educational.

Finally, the educational aspects of robotics are discussed in Stein et al., which focuses on a solution for teaching computational thinking in classrooms. To address the problem of high costs and limitations of physical robots, the authors propose a solution in the form of a networked virtual robotics platform, which is highly accessible for novice students and their teachers. Such platform is used in conjunction with a block-based programming environment, which significantly reduces the barrier to entry. The paper also presents components of a curriculum designed to teach computational thinking skills through robotics programming challenges, including autonomous challenges and in-class competitions. The authors demonstrate the use of this virtual robotics platform in an in-person class during the Spring 2022 semester, showing that students had a significant improvement in both attitudes and aptitudes.

As emphasized in the above mentioned articles, robotic challenges and competitions emerge as an unparalleled avenue for motivating students to apply classroom-acquired knowledge and inspiring researchers to pioneer innovative solutions. These collaborative endeavors, where diverse teams converge to tackle common goals, foster a focused approach to problem-solving. The global proliferation of robot competitions is a testament to their effectiveness in nurturing healthy competition, driving participants towards ingenious solutions, and increasing their wiliness to learn important 21st century skills.

